# Photocatalytic degradation of different types of microplastics by TiO_x_/ZnO tetrapod photocatalysts

**DOI:** 10.1016/j.heliyon.2023.e22562

**Published:** 2023-11-18

**Authors:** Yanling He, Atta Ur Rehman, Muxian Xu, Christelle A. Not, Alan M.C. Ng, Aleksandra B. Djurišić

**Affiliations:** a Department of Physics, The University of Hong Kong, Pokfulam, Hong Kong, China; b Department of Physics & Core Research Facilities, Southern University of Science and Technology, Shenzhen, 518055, China; c Dept. of Earth Science, The University of Hong Kong, Pokfulam, Hong Kong, China; d Department of Physics, Boston College, Chestnut Hill, MA, USA

**Keywords:** Photocatalysis, TiO_2_, ZnO, Microplastics

## Abstract

We investigated the use of titania coated ZnO tetrapods for photocatalytic degradation of two common types of microplastics, namely polyethylene (PE) microparticles and polyester (PES) microfibers. We found that the plastics morphology affects the rate of degradation, and that the use of electron scavengers is needed to maintain the reactivity of the photocatalysts over a prolonged period of time. Complete mass loss of PE and PES is achieved under UV illumination for 480 h and 624 h, respectively. In addition to pristine microplastics, the degradation of environmental microplastics sample (consisting primarily of polypropylene) was also demonstrated, though in this case longer degradation time (∼816 h) was needed to achieve complete mass loss of the samples.

## 1 Introduction

Increased levels of plastics production, combined with their persistence in the environment (natural degradation would take centuries), have resulted in increasing levels of plastics pollution, rising concerns about microplastics (MPs) pollution, and increased interest in possible methods for the remediation of MP pollution [[1], [2], [3], [4], [5], [6], [7], [8], [9], [10], [11], [12]]. The plastics in the environment consist of large plastic pieces (megaplastics, macroplastics, mesoplastics), as well as smaller plastic debris (MPs, with size in the range between 1 μm and 5 mm, and nanoplastics (NPs), with size <1 μm) [1,3]. The MPs consist of primary MPs (manufactured in that size, typically in personal care products or textiles) and secondary MPs (generated from fragmentation of larger plastic waste) [2]. Due to their small size, MPs are a greater environmental concern as they can be easily transported. Consequently, MPs have been detected in air, water, sediments, soil, and living organisms [2,3]. Thus, there is an urgent need for remediation of the MP pollution. Among different remediation methods, various methods for physical removal of MPs (absorption, filtration, etc.) do not actually solve the problem as the MPs are simply transferred elsewhere, for example from water effluent to sludge. The use of sludge as a fertilizer then exacerbates microplastics pollution, since it results in significant deposits of MPs on farmlands (43000–63000 tons per year estimated for Europe [10], illustrating the need for complete removal of MPs from the environment. On the other hand, permanent removal could be achieved by biodegradation, advanced oxidation processes (including photocatalysis), and catalysis [[1], [2], [3],^6^].

Among these processes, photocatalysis is of significant interest as it commonly shows significantly higher degradation efficiency compared to biodegradation and it is performed under milder conditions compared to chemical degradation (catalysis) [6,8]. In addition, photocatalysis can be combined with the collection of plastic waste, as demonstrated on various self-propelled microrobot examples for capture and degradation of MPs [[13], [14], [15], [16]]. Moreover, due to its lack of selectivity, photocatalytic degradation is suitable for degradation of wide range of plastics to smaller plastics fragments, smaller organic molecules, and ultimately CO_2_ [1].

Consequently, photodegradation processes including the effect of illumination and the effects of a number of different photocatalysts on degradation/chemical changes of different plastics have been investigated in recent years [[17], [18], [19], [20], [21], [22], [23], [24], [25], [26], [27], [28], [29], [30], [31], [32], [33], [34], [35], [36], [37], [38], [39], [40], [41], [42], [43], [44]], and the results have been summarized in Table S1. The degraded plastics include polyolefins, such as polyethylene (PE) and polypropylene (PP), polystyrene (PS), polyvinyl chloride (PVC) polyethylene terephthalate (PET), polyamide fibers, and poly(methylmethacrylate) [1,8,9,12]. However, improvements in the photocatalytic performance are needed [1,8]. For example, reported efficiencies for degradation of PE and PP, which represent the most common plastic materials (57 % of total plastics produced) have commonly been below 75 % [1], and the works reporting high degradation efficiency and/or complete mineralization have been scarce [3,7,9] involving TiO_2_ [3,9,20,21] and Nb_2_O_5_ [7,19], and commonly involve UV-C light [20,21]. In addition, the applicability of photocatalytic degradation to actual MPs collected from the environment has not been investigated since the studies investigating “real-world plastics” typically consider virgin consumer plastics [19].

Thus, there is a well-recognized need for the development of novel photocatalysts to improve photocatalytic plastics conversion [1]. This is a challenging task due to stability of plastic materials, in particular saturated carbon backbone plastics, such as PE, which are very stable and thus exhibit high resistance to degradation [2]. There are many possible candidates for photocatalyst materials. Metal oxides, such as ZnO [27,28] and TiO_2_ [[20], [21], [22], [23],[29], [30], [31], [32], [33], [34], [35], [36], [37], [38], [39]], have been among commonly investigated photocatalysts for microplastics degradation [4,7,9]. While complete degradation of PE under UV-C light (254 nm) was reported for TiO_2_, negligible degradation of PE was obtained under UV-A (365 nm) and visible illumination [20]. Thus, to improve photocatalytic degradation of microplastics under more mild conditions compared to UV-C illumination, we have selected photocatalyst based on ZnO instead. ZnO has important advantages over TiO_2_, namely improved crystallinity (which can lead to lower recombination losses) and higher mobility of photogenerated electrons, which can lead to improved photocatalytic activity [45,46]. However, ZnO is less chemically stable compared to TiO_2_ [46]. Chemical stability of ZnO-based photocatalyts can be improved by using core-shell structures [45], with chemically stable shell (TiO_2_) providing an additional advantage of improving charge carrier separation and thus enhancing photocatalytic activity [4,45,47]. For the core material of the ZnO/TiO_2_ photocatalysts, we have selected ZnO tetrapods, due to their exceptional optical properties (low nonradiative recombination losses) [[48], [49], [50]], and consequently high photocatalytic activity (that we have previously demonstrated on dye degradation) [50]. The exceptionally low nonradiative recombination losses of ZnO tetrapods enable them to have high photocatalytic activity, despite their low Brunauer–Emmett–Teller (BET) surface area [50], in agreement with reports that photocatalytic activity [[50], [51], [52]] and reactive oxygen species (ROS) production [53] of ZnO is more significantly determined by its crystallinity, morphology, and/or native defect concentrations, compared to surface area [[50], [51], [52], [53]]. The ZnO tetrapods were coated with TiO_2_ shells using atomic layer deposition (ALD) to ensure conformal coating, and applied to photocatalytic degradation of microplastics. The effect of plastics composition (PE, polyester (PES)) and morphology (particles, fibers) on the degradation process has been investigated. Complete mass loss has been achieved for PE MP particles, PES fibers, and environmental MPs, which predominantly consisted of polypropylene (PP).

## 2 Materials and methods

### 2.1 Materials

TiO_2_ precursor tetrakis (dimethylamido)titanium (TDMAT, 99.999 %) was obtained Suzhou Fornano Electronic Technology Co., Ltd,. Zn powder (99.995 %) and Na_2_S_2_O_8_ were obtained from Sigma Aldrich. Spin trap 5-(Diethoxyphosphoryl)-5-methyl-1-pyrroline-N-oxide (DEPMPO) was purchased from ABCam. PES was obtained from Dingwang Co., Ltd., while PE particles were extracted from the facial scrub (Neutrogena), using previously described procedure [23,36]. Briefly, 60 g of facial scrub was mixed with 500 mL of distilled boiling water, and the warm mixture was filtered through a filter with pore size of 100 μm, repeatedly washed with distilled water, dried at 30 °C for 24 h and stored until use [36]. Following this procedure, the PE spheres with diameters ranging between 200 and 500 μm were extracted from the facial scrub.

Environmental microplastics pieces are retrieved from a water sample collected in Tsing Lung Tau (22.35713, 114.03479) located on the Southwest coast of the New Territories in Hong Kong. The site corresponds to a remote beach in Tsing Lung Tau in a semi-enclosed bay with 2–30 m water depth. Water sample was collected at the water boundary, where the wave was about to break since macro- and micro-plastics accumulated there following the conclusion of Ref. [54]. A total of 50 L surface seawater is filtered by a 0.3 mm sieve. Microplastics present in those 50 L were extracted from water following the modified guidelines given by NOAA Marine Debris Program [55]. Microplastic debris were separated from other material present by 0.1 M KOH digestion and density separation by saturated NaCl solution. Microplastics captured were separated between two size fraction (0.3–1 mm and 1–5 mm) and examined under a microscope. No shape or polymer sorting were done prior to degradation process. PES fibers and environmental plastics samples were ground into particles with size of ∼2 μm for degradation using ball milling (Retsch, PM400).

### 2.2 Synthesis of ZnO and black TiO_2_/ZnO

To prepare ZnO tetrapods, procedures in our previous work [49,50] were followed. Briefly, 200 mg of 99.995 % Zn powder was placed in an alumina crucible in a quartz tube, and the quartz tube was inserted into a horizontal tube furnace. The furnace was heated up to 950 °C and maintained at that temperature for 10 min in the flow of Ar gas, which was bubbled through the water before entering the furnace. Cotton-like ZnO tetrapods were deposited inside the quartz tube downstream from the crucible. After several minutes, the quartz tube was taken out of the furnace and cooled to room temperature. Black titania coating was performed by atomic layer deposition (ALD) deposition, using a Savannah S200, Ultratech ALD chamber equipped with ports for the TDMAT and H_2_O. To prepare a 10 nm black titania coating, ZnO tetrapods were placed in the powder sample holder, and the temperatures of the reactor heater and chamber heater were set to 150 °C and 200 °C, respectively. The TDMAT precursor was heated up to 75 °C to maintain the adequate vapor pressure in the bubbler. Nitrogen was used as purge and carrier gas with a flow rate of 20 sccm during the titanium/O_2_ precursor pulse processes. One cycle consisted of a 0.2 s pulse of TDMAT with 8 s purge time and a 0.01 s pulse of water, with 15 s purge time. The pressure before the deposition was 0.6 Torr.

### 2.3 Characterization

The crystal structures of the photocatalyst samples were examined using X-ray diffraction (Rigaku, Smartlab; operated at 45 kV and 200 mA, Cu Kα source). The morphologies of the photocatalysts and microplastics samples were investigated using transmission electron microscope (TEM) equipped with EDX analyzer (FEI, Tecnai F30 operated at 300 kV), a scanning electron microscope (SEM, NovaNanoSEM, FEI operated at 30 kV), and optical microscope (Leica). The FTIR spectrometer (VERTEX 80v from Bruker) was used for the measurements of the chemical structure of PE, PES and environmental sample before and after photocatalytic degradation, where the spectra were measured on plastics:KBr powder pellets (4 mg plastics/500 mg KBr). The investigation of the *in situ* PE degradation with 2 mg PE mixed with 100 mg TiO_2_/ZnO tetrapod was carried out using IR-AFM (Nano IR2 from Bruker). BET surface area was determined using a Micromeritics ASAP2020 physisorption analyzer with pure nitrogen at 77 K after each sample was evacuated at 573 K for 4 h. ESR measurements were carried out on a Bruker EMXPlus. Different weights of sodium persulfate (100 mg–400 mg）were added into the solution of 50 mg photocatalyst/5 mL de-ionized water. 0.9 mL of the catalyst solution was extracted and mixed with 0.1 mL of DEPMPO with the concentration of 5 mg/mL. The mixture was filled into quartz capillaries for measurements. UV light with light intensity of 480 mW/cm^2^ was used during the measurements.

### 2.4 Photocatalytic degradation of microplastics

The 50 mg of catalyst was dispersed in a Petri dish containing 25 mL of de-ionized water. Before the photocatalysis, the plastics (PE, PES, and environmental sample) were placed in 12 % H_2_O_2_ for 96 h. The FTIR spectra of PE samples before and after H_2_O_2_ pre-treatment are shown in Fig. S1. The purpose of the pre-treatment was to improve the oxidation of the MPs, and a small peak attributed to OH group vibrations in the region of 3300–3600 cm^−1^ [33] is observed, while no other evidence of oxidation is found. For the photocatalytic degradation experiments, initial quantity of MPs (5 or 10 mg, as specified) was added into the solution. 400 mg of Na_2_S_2_O_8_ was added as electron scavenger [56,57], unless specified otherwise. The mixture was stirred using a magnetic stirrer in the darkness to achieve equilibrium and was then placed under a 365 nm UV light (B-100 AP, UVP, 40 mW/cm^2^ or PLS-LED100C, PerfectLight, 480 mW/cm^2^; the effect of illumination power on the degradation is small, as illustrated in Fig. S2, with slightly faster degradation occurring for higher illumination power). Control experiments involved performing the same experiments in the dark. All the photocatalytic tests were performed at room temperature for a set period of time. Plastic degradation was measured by measuring mass loss at fixed time intervals, which is commonly used method to determine degradation of plastics [17,23,33,36,41]. To separate the plastics for mass loss measurements, filter with 2 μm pore size was used, and filtered particles were washed with distilled water 5 times. The separated particles were then dried at 30 °C for 24 h and weighted. Mass loss was calculated using the following equation [23,33,36]: Mass loss (%) = 100 (M_0_–M)/M_0_, where M_0_ is the initial mass of the microplastics and M is the mass at the hours of irradiation.

## 3 Results and discussion

**Fig. 1:**
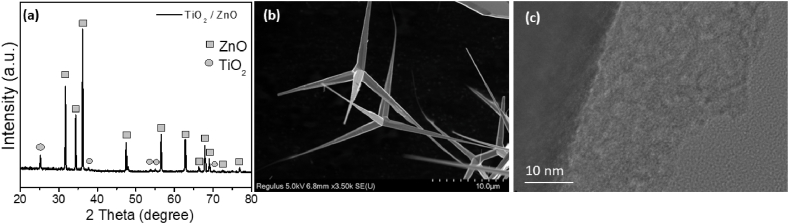
a) XRD patterns of ZnO and ZnO/TiO_2_ photocatalysts. The squares denote peaks corresponding to ZnO (JCPDS 36–1451), while circles denote peaks corresponding to anatase TiO_2_ (JCPDS 01–0562). b) SEM image of ZnO tetrapod. c) TEM image of black titania coating.

To construct a core-shell heterojunction photocatalyst, ZnO tetrapods were selected as the core material due to their exceptionally low defect density and high photocatalytic activity [[48], [49], [50]]. However, bare ZnO tetrapods show lower efficiency for microplastics degradation compared to TiO_2_/ZnO tetrapod core-shell photocatalysts, as shown in Table S2, while UV illumination without photocatalyst (Table S2) resulted in negligible mass loss. Thus, we investigated ZnO tetrapods coated with titania shell deposited by ALD. Fig. 1 shows the XRD patterns and morphology of ZnO tetrapods coated with titania. We can observe that the samples contain peaks corresponding to ZnO and anatase titania (Fig. 1a). The tetrapods morphology can be observed in Fig. 1b, while TEM image (Fig. 1c) shows that the surface of ZnO tetrapods is fully covered with titania coating. This is expected to enhance the stability of ZnO tetrapods, as well as possibly improve photocatalytic activity by facilitating separation of photogenerated charge carriers at ZnO/TiO_2_ interface [58].

Improved charge separation enables lower recombination losses, so that overall photocatalytic activity is enhanced despite lower BET surface area, which is 11.4 m^2^/g for bare tetrapods, and 7.6 m^2^/g for TiO_2_/ZnO core-shell structures (Adsorption/desorption curves for BET measurement are shown in Fig. S3). In the XRD patterns, we can observe strong peaks corresponding to ZnO and weak peaks corresponding to anatase TiO_2_, in agreement with TEM images which show poor crystallinity of TiO_2_, as expected for low temperature ALD growth of disordered titania. We have also tested the photocatalyst activity under simulated solar illumination (Fig. S4), as black titania shell is expected to contribute to photocatalytic activity under visible light illumination. While TiO_2_/ZnO photocatalysts exhibit higher degradation of PE compared to P25 photocatalyst with ALD titania shell, the observed degradation is much slower compared to UV illumination. This is likely due to inferior crystallinity of titania shell, so that improved performance is obtained under UV illumination where the majority of charge carriers are generated within high crystallinity ZnO core. Thus, we will focus on investigating photocatalytic degradation of MPs under UV 365 nm illumination.

**Fig. 2:**
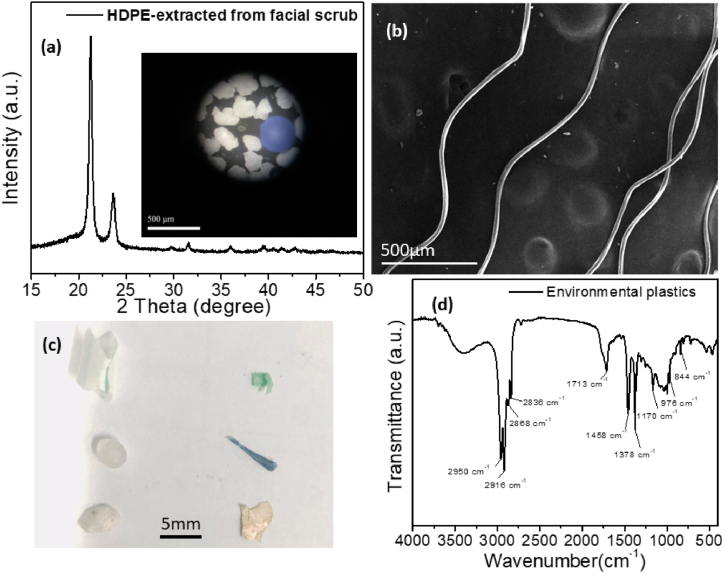
a) XRD patterns of HDPE particles extracted from facial scrub. The inset shows optical microscopy image. b) SEM image of the PES fibers c) photos and d) FTIR spectra of environmental plastics samples measured before any treatment.

Fig. 2a shows the XRD pattern of the high density polyethylene (HDPE) particles, while the inset shows microscopy image of the particles. The obtained results clearly identify the particles as PE, and are in good agreement with previous report [23,36]. In contrast with irregular shape PE particles, PES fibers have width smaller than 100 μm, but they have length in mm range (Fig. 2b). Fibers are a particularly important morphology for MPs, since microfibers are considered most important type of MP in terms of prevalence and ecotoxicity [21]. Environmental MPs consist of significantly larger pieces, as shown in Fig. 2c. One piece was selected for degradation (shown in the lower right corner), and its composition was examined by FTIR, as shown in Fig. 2d. The FTIR spectra closely resemble those of degraded PP [33]. In addition to characteristics peak observed in pure PP due to C–C and C–H vibrations at ∼2950 cm^−1^, ∼2918 cm^−1^, ∼2866 cm^−1^, 2837 cm^−1^, 1454 cm^−1^, 1375 cm^−1^, 1166 cm^−1^, and ∼973 cm^−1^ [33], we also observe the broad peak due to O–H group vibrations in the region 3300-3600 cm^−1^, peak corresponding to carbonyl group in the region 1650-1750 cm^−1^ [33,59], and a number of small peaks in the range 1200-900 cm^−1^, likely corresponding to carbon-oxygen bonds [59], similar to observations in FTIR spectra of photocatalytically degraded PP [33] and/or weathered PP [59]. The observed changes, namely the appearance of vibrations corresponding to hydroxyl and carbonyl vibrations, are consistent with changes observed in FTIR spectra of PE and PP samples subjected to weathering [59]. Thus, environmental samples have obviously undergone chemical changes due to weathering, as expected from samples collected from the water boundary of a beach where they have been exposed to weather for unknown time.

**Fig. 3:**
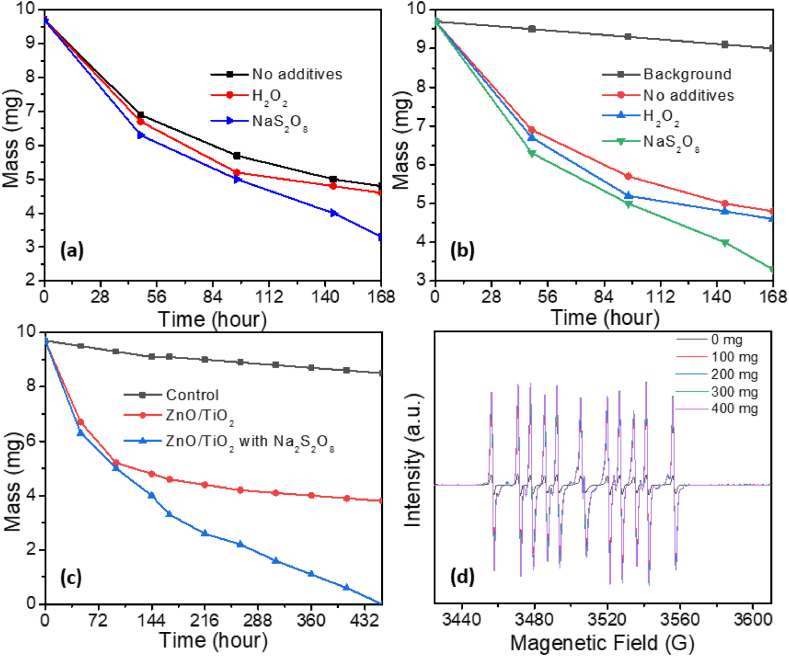
a) PE degradation by TiO_x_/ZnO with no additives, H_2_O_2_ and electron scavenger; b) PES degradation by TiO_x_/ZnO with no additives, H_2_O_2_ and electron scavenger; c) PE long time degradation with TiO_x_/ZnO photocatalyst with/without electron scavenger. d) ESR spectra with DEPMPO spin trap for different amount of Na_2_S_2_O_8_. Illumination power was 40 mW/cm^2^.

**Fig. 4:**
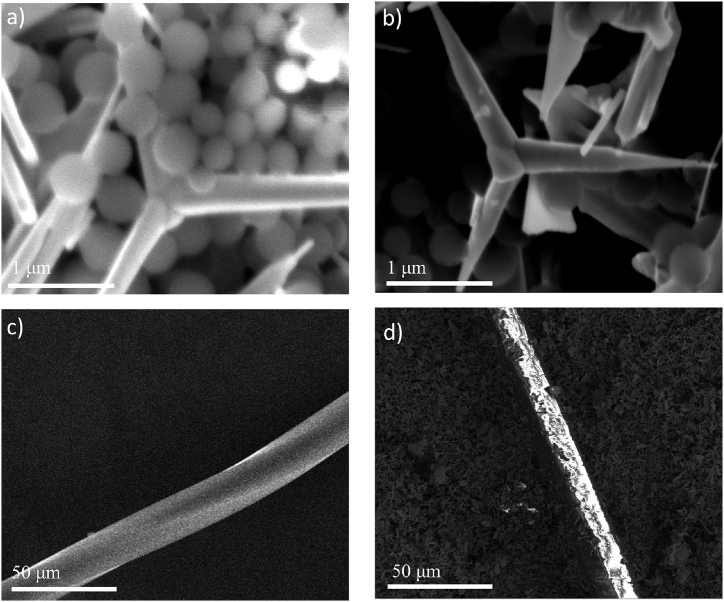
a) SEM image of PE on TiO_x_/ZnO before reaction; b) SEM image of PE on TiO_x_/ZnO after 48 h reaction with Na_2_S_2_O_8_; c) SEM image of PES on TiO_x_/ZnO before reaction; d) SEM image of PES on TiO_x_/ZnO after 96 h reaction with Na_2_S_2_O_8._

To investigate photocatalytic activity of TiO_2_/ZnO photocatalyst, we have first conducted photocatalytic degradation on PE and PES, as shown in Fig. 3. We investigated the effect of H_2_O_2_ and Na_2_S_2_O_8_, as it is known that the addition of electron scavengers such as hydrogen peroxide and sodium persulfate can enhance photocatalytic activity by preventing recombination of photogenerated charge carriers [56]. In addition, Na_2_S_2_O_8_ can generate longer lived sulfate radicals [57]. We can observe that H_2_O_2_ has negligible effect on photocatalytic degradation of both MPs, while increased degradation can be observed with the addition of Na_2_S_2_O_8._ For both PE and PES we can see saturation of the degradation after some time, similar to some previous reports [28]. With the addition of Na_2_S_2_O_8_, PE can be 100 % mass loss can be achieved. From ESR spectra shown in Fig. 3d, we can observe that the addition of Na_2_S_2_O_8_ results in significant enhancement of the ROS generation, as expected since DEPMPO is capable of forming adducts with both OH^•^ radicals and OH^•^ formed by nucleophilic substitution of SO_4_^•-^ radicals [60]. We can also observe that while degradation for PES is also accelerated with the addition of Na_2_S_2_O_8_, it still saturates eventually. To try to understand the observed differences in behavior of PE and PES, we examined the morphology of the MP/photocatalyst mixture initially and after specified time of photocatalytic degradation, as shown in Fig. 4. In the case of PE, we can observe reduction in particle sizes and disappearance of the particles. For PES fibers, we can observe a change in morphology indicating that degradation occurs, but mass loss trend is slowing down over time. Previous report on high percentage of degradation (97 %) of polyamide microfibers used UV-C illumination (only ∼6 % mass loss was obtained for UV-A illumination), which is considerably more harsh condition compared to 365 nm. Thus, it is of interest to investigate if high degradation can be achieved under this milder condition. Since it is known that smaller size MPs can be degraded more efficiently [26], we examined the effect of ball milling before degradation.

**Fig. 5:**
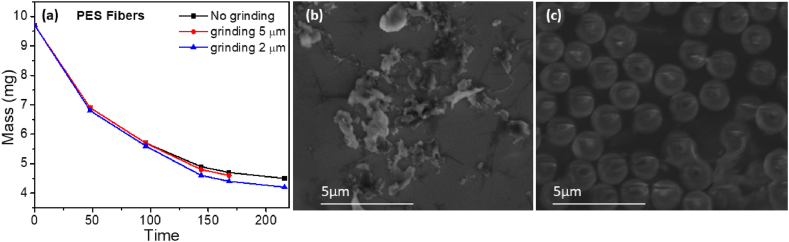
a) Effect of ball milling on the photocatalytic degradation of PES fibers. Experiment was performed with 400 mg Na_2_S_2_O_8_ and under 365 nm UV illumination with intensity 480 mW/cm^2^ b),c) SEM images of PES fibers and environmental MPs after ball milling.

Fig. 5a shows that a reduction in size does solve the problem and that degradation continues. From Fig. 5b, fiber fragments have irregular shapes but much smaller size, which allows them better interaction with photocatalyst. Ball milling of environmental MPs results in particles with more regular shape and size ∼2 μm, as shown in Fig. 5c.

**Fig. 6:**
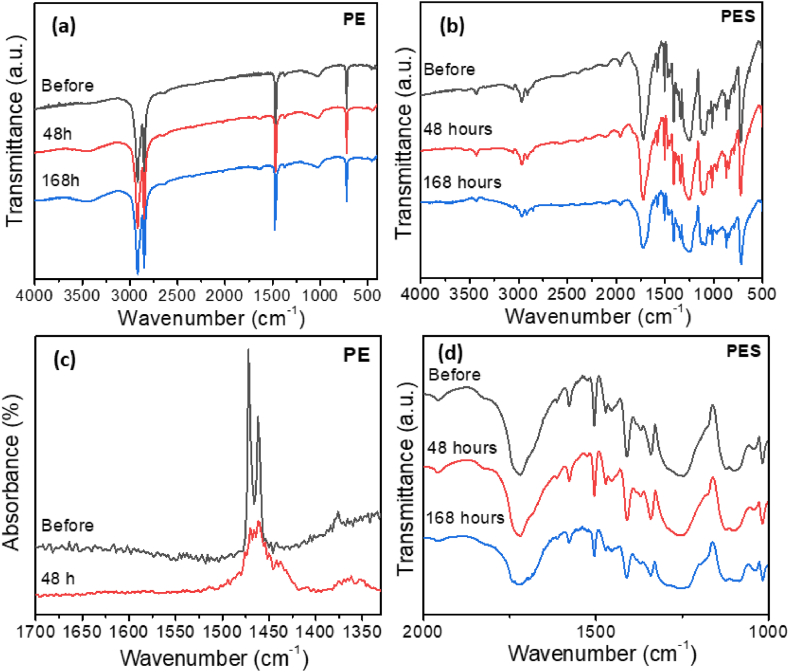
FTIR spectra before and after degradation for a) PE and b) PES; c) IR-AFM spectra of the absorption peak for CH_2_ bond of the PE microplastics for different illumination times; d) FTIR spectra of PES microfibers after 0 h and 168 h of UV illumination in the region 1000-2000 cm^−1^. Degradation was performed under 365 nm UV illumination with illumination power 40 mW/cm^2^.

To further investigate degradation process, FTIR measurements were performed, as shown in Fig. 6. It is known that the dominant reaction during photodegradation of plastics is photooxidation, and that the generation of ROS, though rarely studied [20,[22], [23], [24], [25], [26]], plays a role in the degradation process [18,22]. The ROS in aqueous solutions containing photocatalyst are generated from reactions of photogenerated electrons and holes with O_2_ and OH^−^, and produced superoxide ion and hydroxyl radicals can then generate other reactive species such as H_2_O_2_ [22]. It was proposed that photogenerated carriers (both electrons and holes), and hydroxyl radicals play a key role in photocatalytic degradation of HDPE, while superoxide ion radicals had a minor role based on the experiments with scavengers for different ROS [22], although other studies found that the contribution of different ROS to the degradation process is dependent on the environment (air, water, dissolved salt) [40]. Hydroxyl radicals were proposed to initiate oxidative degradation of PE by creating various polyethylene alkyl radicals [17]. PE alkyl radicals generated by ROS can then react with oxygen to produce peroxyl radicals [17,23,41,42,44]. The reaction propagates, generating new radicals [17,23,[41], [42], [43], [44]], and eventually terminates when two radicals combine to generate inert products [17,41] or react with oxygen [43], producing carboxylates, aldehydes, and ketones [17,41,43]. The reaction intermediates are ultimately oxidized to CO_2_ and H_2_O by the produced ROS [43]. High availability of hydroxyl radicals was proposed to have a key role in the degradation [17], in agreement with observing enhancement of photocatalytic degradation with the addition of Na_2_S_2_O_8_ which significantly increased generation of ROS. The observed changes in the FTIR spectra are in agreement with expectations of oxidative reactions initiated by ROS, although for different types of plastics we can observe differences in three main indicators of oxidation reactions (hydroxyl, carbonyl, and C–O vibrations [59]). The FTIR spectra of extracted HDPE particles is in good agreement with the literature report [23,36]. We can observe characteristic vibrations at ∼2925, 2840, 1467, and 720 cm^−1^ corresponding to vibrations of CH_2_ and CH groups in long alkyl chains [23,36]. Main observation in the FTIR spectra of HDPE is the reduction of characteristic vibrations in FTIR spectra (Fig. 6a), in agreement with the previous report [36]. It should be noted that FTIR is not a quantitative measurement, and we mainly look at the appearance of new peaks (such as for example O–H vibrations and carbonyl group vibrations), or changes in relative ratios of different peaks. While the reduction in intensity of characteristic vibrations of a specific plastic can serve as a possible indicator of degradation, the degradation should also be checked by other methods. The obtained indication of degradation is in agreement with the observed mass loss, degradation observed in SEM images, as well as IR-AFM spectra (Fig. 6c), which was performed *in situ*. We can also observe the appearance of very small peak at ∼1720 cm^−1^, likely corresponding to carbonyl group vibrations [5,33]. For PES microfibers, the observed FTIR spectra before degradation shown in Fig. 5b are consistent with previous reports on polyester/polyethylene terephthalate microplastics [[61], [62], [63]]. After 48 h of degradation, similar to PE, no significant changes in the FTIR spectra can be observed. After 168 h of photocatalytic degradation, we can observe the change of the shape of the peaks in the spectral ranges 1700-1760 cm^−1^ and 1200-1300 cm^−1^, where vibrations of oxygenated groups are expected [59,64], as shown more clearly in enlarged relevant part of the spectrum in Fig. 6d. However, different from PE, broad peak in the range 3100–3600 cm^−1^ cannot be observed. This indicates that while oxidative reactions occur for both types of microplastics, the exact chemistry of photocatalytic degradation is dependent on the type of microplastics being degraded.

**Fig. 7:**
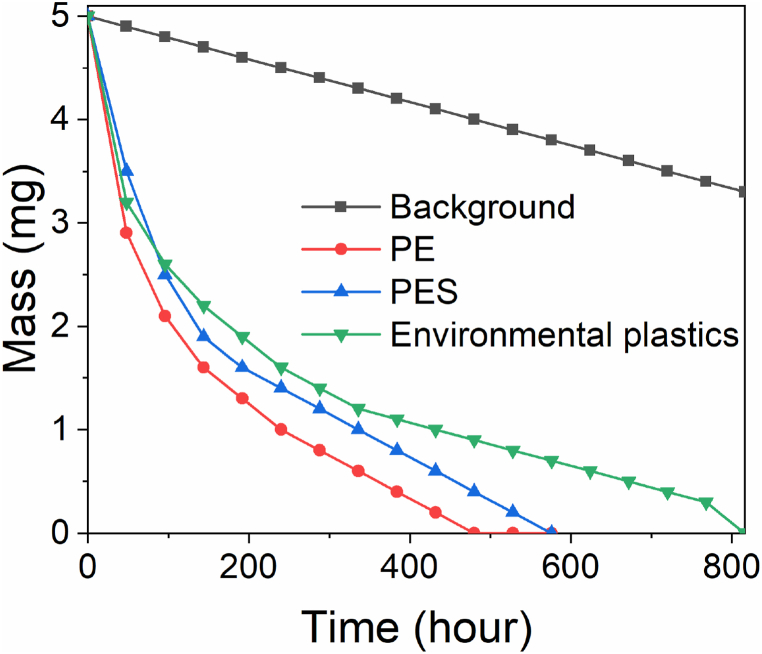
Degradation of different MPs by TiO_2_/ZnO photocatalyst. The experiment was performed with 400 mg Na_2_S_2_O_8_ and under 365 nm UV illumination with intensity 480 mW/cm^2^.

Finally, as the TiO_2_/ZnO catalysts demonstrated good performance in degrading MPs with different chemical composition and morphology (provided that ball milling is performed to ensure suitably small size for efficient mixing with tetrapod photocatalyst), we investigated the photocatalytic degradation of environmental MPs. Obtained results are shown in Fig. 7. It can be observed that complete mass loss can be obtained for all types of MPs investigated, but the degradation time varies for different materials. This illustrates suitability of TiO_2_/ZnO core-shell catalysts for photocatalytic degradation of microplastics. It should also be noted that complete mass loss does not necessarily imply complete mineralization, but it is expected that any degradation products in solution (NPs, polymer fragments, other organic degradation products such as oxygenated small low molecular weight fragments (aliphatic carboxylic acids, alcohols, aldehydes, and ketones) [17,41,43]), would eventually degrade into main product of photocatalytic degradation of different plastics, namely CO_2_ [19] since the same mechanisms (generation of ROS, followed by reaction of pollutant with photogenerated carriers and/or ROS and pollutant degradation) are generally applicable to organic pollutants [17]. In addition, smaller plastics particles degrade faster than larger ones [17] and thus NPs should degrade much faster than MPs, while smaller organic molecules are also expected to degrade fast compared to MPs, based on previous reports on the use of ZnO tetrapods for photocatalytic degradation of organic compounds [49,50,53]. The use of specially designed reactors which can facilitate the analysis of produced gas [19] and/or HPLC to analyze reaction intermediates would be useful in future work, for example to compare the degradation of virgin and weathered plastics.

## 4 Conclusions

In summary, we demonstrated efficient photocatalytic degradation of different MPs, namely HDPE extracted from facial scrub, PES microfibers, and environmental MPs using TiO_2_/ZnO photocatalyst under 365 nm illumination. The degradation was found to be strongly dependent on the morphology of MPs, since the fibers exhibited saturation of the mass loss, with observable damage to the fibers but still maintaining the fiber integrity. The achievement of 100 % mass loss of MPs required ball milling to obtain sufficiently small fragments (∼2 μm particles) that can be efficiently degraded for MPs with large size (PES fibers, environmental plastics pieces), as well as the addition of an electron scavenger Na_2_S_2_O_8_ to significantly enhance the generation of ROS and thus enhance the photocatalytic degradation. Although different treatment time was needed for different types and morphologies of MPs, 100 % mass loss could be achieved for all materials investigated.

## CRediT authorship contribution statement

**Yanling He:** Investigation, Methodology, Visualization. **Atta Ur Rehman:** Investigation, Methodology, Visualization. **Muxian Xu:** Investigation. **Christelle A. Not:** Resources, Writing – review & editing. **Alan M.C. Ng:** Funding acquisition, Project administration, Resources, Supervision, Conceptualization, Writing – review & editing. **Aleksandra B. Djurišić:** Conceptualization, Funding acquisition, Project administration, Resources, Writing – original draft, Writing – review & editing.

## Declaration of competing interest

The authors declare that they have no known competing financial interests or personal relationships that could have appeared to influence the work reported in this paper.
