# Supplementary material for: Photocatalytic degradation of different types of microplastics by TiOx/ZnO tetrapod photocatalysts
Source: Heliyon. 2023 Nov 18;9(11):e22562. doi: 10.1016/j.heliyon.2023.e22562 (PMC10687295; doi:10.1016/j.heliyon.2023.e22562)
Supplement: Multimedia component 1 [file mmc1.pdf]

Supporting Information

Photocatalytic degradation of different types of microplastics by TiO_x/ZnO tetrapod photocatalysts

Yanling He,[1] Atta Ur Rehman,[1] Muxian Xu,[2] Christelle A. Not,[3] Alan M. C. Ng[2,*] Aleksandra B. Djurišić,[1,*]

[1]Department of Physics, The University of Hong Kong, Pokfulam, Hong Kong SAR, China.

[2]Department of Physics & Core Research Facilities, Southern University of Science and Technology, Shenzhen, 518055, China.

[3] Dept. of Earth Science, The University of Hong Kong, Pokfulam, Hong Kong SAR, China.

E_mail: dalek@hku.hk; ngamc@sustech.edu.cn

Table S1. Summary of literature reports on photocatalytic degradation of MPs. CI denotes carbonyl index.

Photocatalyst	Plastics	Light	% Mass loss	Time [h]	Ref.
ZnO	LDPE	Visible (halogen lamp)	Unknown, 30% increase in CI	175	1.
Cu_2O	PS	Visible (LED light)	23% conc. Reduction, 15% mineralization	50	2.
TiO_2	PE film	Simulated solar (Xe lamp)	Unknown, morphology, properties change	4	3.
TiO_2	PP MPs	Simulated solar (Xe lamp)	Unknown, properties change	4	4.
C,N-TiO_2	HDPE MPs	Visible (LED light)	72%	50	5.
KPF_6/BiOBr	PE pieces	Visible (LED light)	6.52%	30	6.
Carbonized TiO_2	PP globules	UV-C	5.622%	400	7.
TiO_2	PS MPs	UV-A	Unknown, particle size reduction (21% diameter reduction)	0.3	8.
N-TiO_2	HDPE	Visible, fluorescent	2.86%	8	9.
TiO_2, TiO_2-RGO	PP film	Sunlight	Unknown, morphology change	>500	10.
CuMgAlTi-LDH	PS MPs	Simulated solar (Xe lamp)	Unknown, particle size reduction 54%	300	11.
TiO_2	5 µm PS spheres	254 nm UV-C	100%	12	12.
C,N-TiO_2	HDPE MPs	Visible, LED light	75.44%	50	13.
TiO_2	Polyamide 66	UV-C	97%	48	14.
NH_2-MIL-88B(Fe)/ MoS_2	HDPE	Visible light, Xe lamp with filter	Unknown, fragmentation observed	48	15.
ZnO	PP	Visible,tungsten-halogen	65% volume reduction	456 h	16.
BiOCl	PE, PP, Nylon	Visible, Xe lamp with filter	Unknown, morphology change, fragmentation, CI increase	10	17.
B-goethite	PE film composite	UV light	12.6	300	18.
NiO_x	PE film composite	Solar	33	240	19.
α-FeOOH	PE, PP	Xe lamp	81.89 (PP), ~50 (PE)	480	20.
α-Fe_2O_3	PE,PP	Xe lamp	67.47 (PP), ~35 (PE)	480	20.
$NiAl_2O_4$	PE	Visible, metal halide lamp	12.5	5	21.
TiO_2/ZnO	PE, PES	365 nm UV light	100%	480 (PE), 624 (PES)	This work

Table S2. Degradation of different MPs under UV illumination (365 nm, 40 mW/cm^2) by ZnO tetrapods and TiO_2/ZnO tetrapods.

	Catalyst	Degraded weight (%) (48 hours)
**Polyethylene**	No photocatalyst	**2%**
	ZnO Tetrapod	**25%**
	10 nm TiO_2 / ZnO Tetrapod	**31%**
**Polyester**	No photocatalyst	**2%**
	Tetrapod ZnO	**17%**
	10 nm TiO_2 / ZnO Tetrapod	**21%**

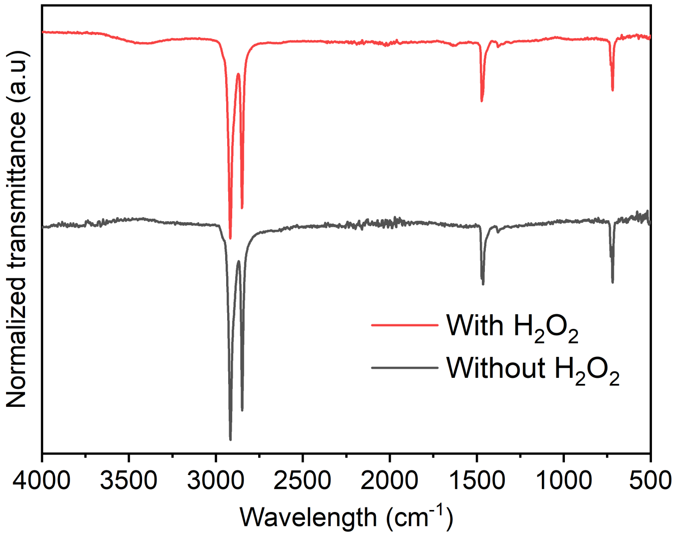

Figure S1. FTIR spectra of PE MPs before (black line) and after (red line) H_2O_2 pre-treatment.

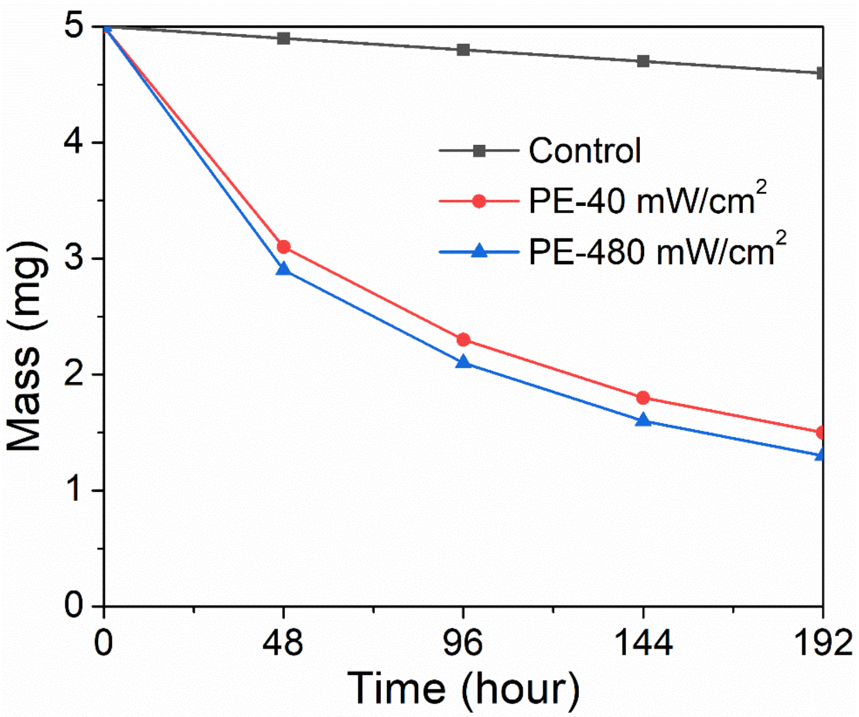

Figure S2. Mass loss of PE MPs by ZnO/TiO_2 photocatalysts under 365 nm UV illumination at different illumination powers (40 mW/cm^2 and 480 mW/cm^2).

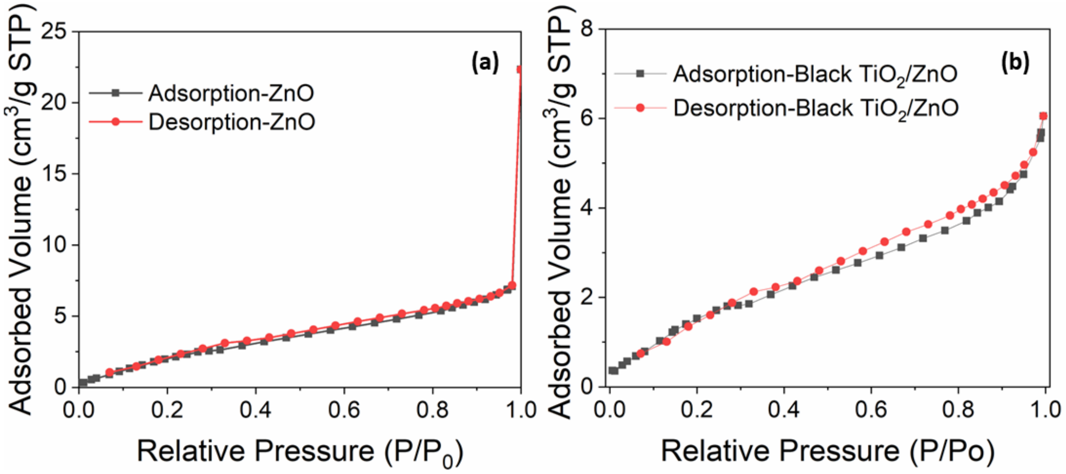

Figure S3. Adsorption/desorption curves of ZnO and ZnO/TiO_2.

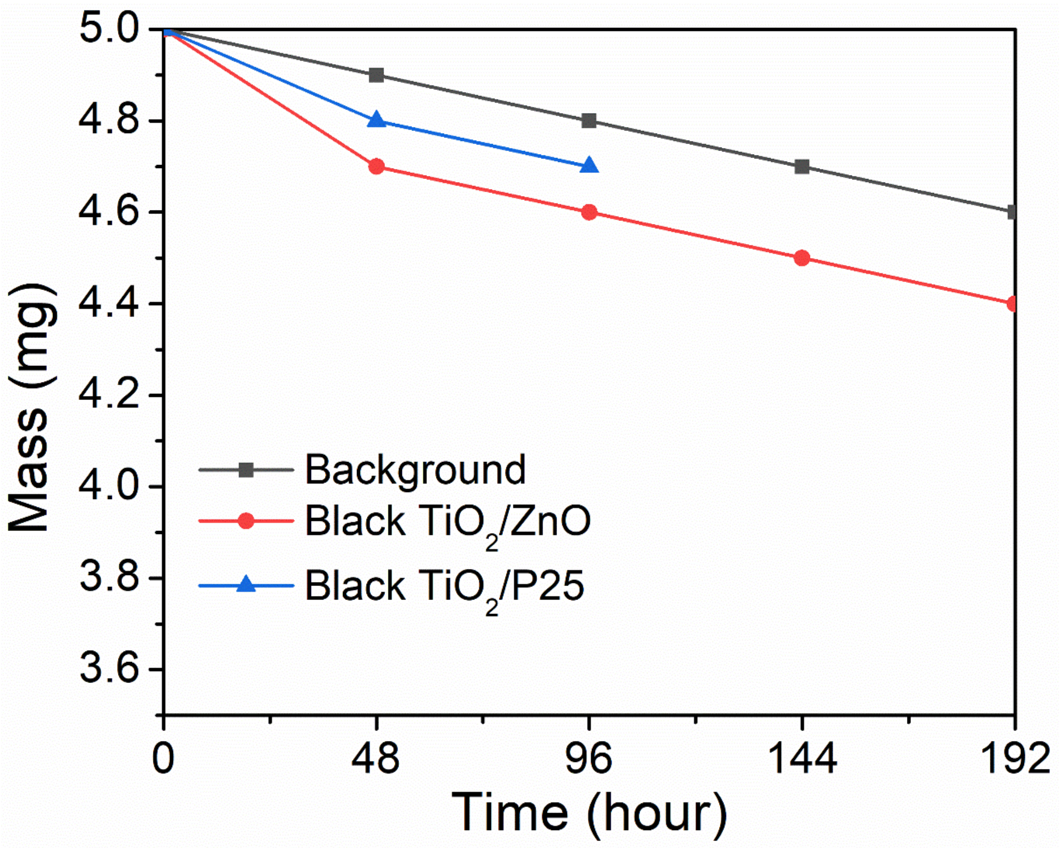

Figure S4. Mass loss of PE MPs under simulated solar illumination (at a power of 300 mW/cm^2) with different core-shell photocatalysts.

References

[1] Tofa, T. S., Kunjali, K. L., Paul, S., & Dutta, J. (2019). "Visible light photocatalytic degradation of microplastic residues with zinc oxide nanorods. Environ." Chem. Lett, 17(3): 1341-1346.

[2] Acuña-Bedoya, J. D., Luévano-Hipólito, E., Cedillo-González, E. I., Domínguez-Jaimes, L. P., Hurtado, A. M., & Hernández-López, J. M. (2021). "Boosting visible-light photocatalytic degradation of polystyrene nanoplastics with immobilized Cu_xO obtained by anodization." J. Environ. Chem. Eng., 9(5): 106208.

[3] Luo, H., Xiang, Y., Tian, T., & Pan, X. (2021). "An AFM-IR study on surface properties of nano-TiO_2 coated polyethylene (PE) thin film as influenced by photocatalytic aging process." Sci. Total Environ., 757: 143900.

[4] Luo, H., Xiang, Y., Li, Y., Zhao, Y., & Pan, X. (2021). "Photocatalytic aging process of Nano-TiO_2 coated polypropylene microplastics: Combining atomic force microscopy and infrared spectroscopy (AFM-IR) for nanoscale chemical characterization." J. Hazard. Mater., 404: 124159.

[5] Vital-Grappin, A. D., Ariza-Tarazona, M. C., Luna-Hernández, V. M., Villarreal-Chiu, J. F., Hernández-López, J. M., Siligardi, C., & Cedillo-González, E. I. (2021). "The Role of the Reactive

Species Involved in the Photocatalytic Degradation of HDPE Microplastics Using C,N-TiO_2 Powders." Polymers, 13(7): 999.

[6] Du, C., Feng, W., Nie, S., Zhang, J., Liang, Y., Han, X., Wu, Y., Feng, J., Dong, S., Liu, H., & Sun, J. (2021). "Harnessing efficient in-situ H_2O_2 production via a KPF_6/BiOBr photocatalyst for the degradation of polyethylene." Sep. Purif. Technol., 279: 119734.

[7] Saifuddin, M., Ghaffari, Y., Park, S. Y., & Kim, C. G. (2022). "Rapid surface degradation of co-axially arranged polypropylene globules by nanoporous carbonized TiO_2 assisted with UV-C." Environ. Res, 212: 113422

[8] Kim, H., Kwon, H., Song, R., Shin, S., Ham, S.-Y., Park, H.-D., Lee, J., Fischer, P., & Bodenschatz, E. (2022). "Hierarchical optofluidic microreactor for water purification using an array of TiO_2 nanostructures." npj Clean Water, 5(1): 62.

[9] Ariza-Tarazona, M. C., Villarreal-Chiu, J. F., Barbieri, V., Siligardi, C., & Cedillo-González, E. I. (2019). "New strategy for microplastic degradation: Green photocatalysis using a protein-based porous N-TiO_2 semiconductor." Ceram. Int., 45(7, Part B): 9618-9624.

[10] Verma, R., Singh, S., Dalai, M. K., Saravanan, M., Agrawal, V. V., & Srivastava, A. K. (2017). "Photocatalytic degradation of polypropylene film using TiO_2-based nanomaterials under solar irradiation." Mater. Des., 133: 10-18.

[11] Jiang, S., Yin, M., Ren, H., Qin, Y., Wang, W., Wang, Q., & Li, X. (2023). "Novel CuMgAlTi-LDH Photocatalyst for Efficient Degradation of Microplastics under Visible Light Irradiation." Polymers, 15(10): 2347.

[12] Nabi, I., Bacha, A. U., Li, K., Cheng, H., Wang, T., Liu, Y., Ajmal, S., Yang, Y., Feng, Y., & Zhang, L. (2020). "Complete Photocatalytic Mineralization of Microplastic on TiO(2) Nanoparticle Film." iScience, 23(7): 101326.

[13] Ariza-Tarazona, M. C., Villarreal-Chiu, J. F., Hernández-López, J. M., Rivera De la Rosa, J., Barbieri, V., Siligardi, C., & Cedillo-González, E. I. (2020). "Microplastic pollution reduction by a carbon and nitrogen-doped TiO_2: Effect of pH and temperature in the photocatalytic degradation process." J Hazard Mater, 395: 122632.

[14] Lee, J.-M., Busquets, R., Choi, I.-C., Lee, S.-H., Kim, J.-K., & Campos, L. C. (2020). "Photocatalytic Degradation of Polyamide 66; Evaluating the Feasibility of Photocatalysis as a Microfibre-Targeting Technology." Water, 12(12): 3551.

[15] Feng, X., Long, R., Liu, C., & Liu, X. (2022). "Visible-light-driven removal of tetracycline hydrochloride and microplastics (HDPE) by nano flower hybrid heterojunction NH_2-MIL-88B(Fe)/MoS_2 via enhanced electron-transfer." Sep. Purif. Technol., 302: 122138.

[16] Uheida, A., Mejía, H. G., Abdel-Rehim, M., Hamd, W., & Dutta, J. (2021). "Visible light photocatalytic degradation of polypropylene microplastics in a continuous water flow system." J. Hazard. Mater., 406: 124299.

[17] Jiang, R., Lu, G., Yan, Z., Liu, J., Wu, D., & Wang, Y. (2021). "Microplastic degradation by hydroxy-rich bismuth oxychloride." J. Hazard. Mater., 405: 124247.

[18] Liu, G., Zhu, D., Zhou, W., Liao, S., Cui, J., Wu, K., & Hamilton, D. (2010). "Solid-phase photocatalytic degradation of polystyrene plastic with goethite modified by boron under UV–vis light irradiation." Appl. Surf. Sci., 256(8): 2546-2551.

[19]Olajire, A. A., & Mohammed, A. A. (2020). “Green synthesis of nickel oxide nanoparticles and studies of their photocatalytic activity in degradation of polyethylene films.” Adv. Powder Technol., 31(1): 211-218.

[20]Ding, L., Guo, X., Du, S., Cui, F., Zhang, Y., Liu, P., Ouyang, Z., Jia, H., & Zhu, L. (2022). “Insight into the Photodegradation of Microplastics Boosted by Iron (Hydr)oxides.” Environ. Sci. Technol., 56(24): 17785-17794.

[21]Venkataramana, C., Botsa, S. M., Shyamala, P., & Muralikrishna, R. (2021). “Photocatalytic degradation of polyethylene plastics by $NiAl_2O_4$ spinels-synthesis and characterization.” Chemosphere, 265: 129021.
